# TET3 as a non-invasive screening tool for the detection of fibrosis in patients with chronic liver disease

**DOI:** 10.1038/s41598-023-33564-7

**Published:** 2023-04-19

**Authors:** Lin-Lin Feng, Ran-Yang Liu, Kun An, Shuang Tang, Jun Wu, Qin Yang

**Affiliations:** 1 Academic Research, Department of Pathology and Pathophysiology, College of Basic Medical Sciences, Guizhou Medical University, Guiyang, 550025 Guizhou Province China; 2 Clinical Laboratory Center, Affiliated Hospital of Guizhou Medical University, Guiyang, 550004 Guizhou Province China; 3 Guizhou Provincial Key Laboratory of Pathogenesis and Drug Research On Common Chronic Diseases, Guizhou Medical University, Guiyang, 550025 Guizhou Province China; 4 Hepatopathy Laboratory, Affiliated Hospital of Guizhou Medical University, Guiyang, 550004 Guizhou Province China

**Keywords:** Biomarkers, Gastroenterology, Hepatology

## Abstract

Ten-eleven translocation protein 3 (TET3) is one of the key enzymes in DNA demethylation which can be expressed in liver tissues. However, the clinical value of TET3 for diagnosis and treatment of chronic liver disease have not been reported previously. We investigated the diagnostic accuracy of serum TET3 as a non-invasive screening tool for liver fibrosis. 212 patients with chronic liver disease from were enrolled in this study. Enzyme-linked immunosorbent assay was used to measure the serum levels of TET3. Receiver operating characteristics (ROC) were determined to examine the diagnostic accuracy of TET3 and combination model for diagnosis fibrosis. Serum TET3 level in fibrosis cases was significantly higher than that in non-fibrosis and controls, respectively. The areas under the ROC curve of the TET3 and fibrosis-4 index for liver fibrosis were 0.863 and 0.813, and 0.916 and 0.957 for liver cirrhosis. The combination of TET3 and fibrosis-4 index had a highly promising positive predictive value for detecting liver fibrosis and cirrhosis different stages of (93.5% and 100%) as compared with each diagnostic tool alone. TET3 is related to the development of liver fibrosis and cirrhosis. The TET3-fibrosis-4 model enhances discriminatory power and represents a promising non-invasive tool for the diagnosis and screening of liver fibrosis.

## Introduction

Chronic liver disease (CLD) creates a major worldwide burden with increasing rates of morbidity and mortality^1,2^. CLD results in approximately 2 million deaths worldwide each year, including one million deaths from complications of cirrhosis and one million deaths from viral hepatitis and hepatocellular carcinoma. The ultimate impact of CLD is affected by a variety of etiologies such as viral infections, alcoholism, fatty liver disease and autoimmune diseases. In North America and Europe, alcohol-related liver disease (ALD), hepatitis C virus infection (HCV) and non-alcoholic fatty liver disease (NAFLD) are more common^3,4^. The predominant etiologies in Asia and Africa are hepatitis B virus infection, HCV, NAFLD and ALD^5^. Regardless of the etiology, the long-term and repeated chronic damage and abnormal repair of hepatocytes and tissues will lead to the deposition of many collagen fibers and fibrosis. Liver fibrosis is an important progressive stage of CLD. If liver fibrosis is not treated in time, it will further deteriorate into liver cirrhosis; some cases of liver cirrhosis will even evolve into liver cancer, thus exerting a serious effect on a patient’s quality of life; it may even threaten life itself.

The progression of liver fibrosis to cirrhosis is not linear; rather, this is a dynamic process that is regulated by age, gender and genetic predisposition; in turn, these factors can interact with a range of environmental factors (e.g., poor diet, alcohol consumption and smoking) to determine the phenotype of patients with chronic liver injury. Recent studies have shown that DNA methylation plays an important role in the occurrence and development of liver diseases^6–8^. DNA methylation refers to that under the action of DNA methyltransferases (DNMTs); the hydrogen atom (5-C) on the fifth carbon atom of CpG dinucleotide cytosine is replaced by a methyl group, thus forming 5-methylcytosine^9^. However, the methylation of cytosine is a dynamic reversible process, and 5-methylcytosine can also undergo a demethylation process that is mediated by ten-eleven translocation (TETs)^10^. TETs can oxidize 5-methylcytosine (5mC) to 5-hydroxymethylcytosine (5hmC) which can then further oxidize to 5-formyl cytosine and 5-carboxyl cytosine, ultimately forming unmodified cytosine through active or passive demethylation mechanisms, thus leading to DNA demethylation and gene activation^11–14^. The TETs are an α-ketoglutarate/Fe-dependent dioxygenase family, containing three members, TET1, TET2 and TET3^15^. Of these, TET3 is a multi-subunit protein with a molecular weight of 180-230kD; the encoding gene is located at 2p13^16^. TET3 has significant tissue specificity in terms of expression^17^ and is mainly expressed in nerve cells^18^, muscle, adrenal gland, and liver^19^. However, whether TET3 is expressed in peripheral blood and can be detected by serological methods has yet to be reported.

In the search for a non-invasive approach to diagnose liver fibrosis, we investigated serum levels of TET3 in CLD patients at Guizhou Medical University and Affiliated Hospitals (Gui Yang, China). Combined with the disease course, we also analyzed the levels of aspartate aminotransferase (AST) and alanine aminotransferase (ALT), along with the fibrosis-4 (FIB-4) index and other indicators to investigate the clinical correlation between TET3 and liver disease. Receiver operating characteristic (ROC) curve analysis^20^ was used to further explore the clinical diagnostic value of TET3 in chronic liver fibrosis by bivariate combined analysis.

## Materials and methods

### Study subjects

A total of 212 patients with chronic liver disease who had undergone cutaneous liver puncture biopsy were enrolled in this study from the Affiliated Hospital of Guizhou Medical University (Gui Yang, China) between December 2020 and March 2022. We recruited 79 males and 61 females with an age range of 15–86 years. There were 38 patients in the non-fibrosis group, including 17 males and 21 females (age range: aged 15–64 years); 102 patients in the fibrosis group, including 62 males and 40 females (age range: 20–86 years) and 72 patients with cirrhosis, including 55 males and 17 females (age range: 19–74 years).

### Inclusion and exclusion criteria

Patients were included if they had (1) a history of chronic hepatitis for more than 6 months, (2) ALT levels that were consistently higher than twice the normal value, and (3) imageological examination. The diagnostic criteria for liver fibrosis were in accordance with the Consensus on Diagnosis and Treatment of liver fibrosis^21^ and was based on chronic hepatitis, combining clinical symptoms, serum testing, imaging and liver biopsy all met the diagnosis for liver fibrosis. Liver biopsy was obtained by percutaneous biopsy with a 16G puncture needle under ultrasound guidance, histological analysis was performed by two senior pathologists in every center, and if the staging is different, the film is re-read to reach a consensus. All the pathologists were blinded to the clinical information. The liver fibrosis was staged by the Metavir system^22^. The diagnostic criteria for cirrhosis were based on a clear history of chronic liver disease; portal hypertension, fundus or esophageal varices, ascites and/or splenomegaly; albumin deficiency; an extended prothrombin time extended; and imaging examination suggested cirrhosis, or liver biopsies were performed in case of ambiguity.

We did not perform pathological staging of hepatitis and liver fibrosis for the following reasons: (1) There was no difference in TET3 levels between hepatitis patients and healthy controls. And the level of TET3 was not related to hepatocyte destruction. (2) 102 patients with liver fibrosis were obtained from liver biopsy, whose pathological stages were concentrated in S1 and S2 stage, and very few were in S3 stage (n = 75, 18, 9, respectively). Therefore, we fused the cases of liver fibrosis stages S1-3 together and roughly divided them into s0 hepatitis, liver fibrosis stage S1-3, and cirrhosis stage S4.

We excluded patients who had advanced stages of liver cancer or those suffering from serious heart, brain, kidney and other internal diseases or immune system disorders. We also excluded patients who were pregnant or lactating, those that had hematopoietic diseases, those with mental illness, and those with HIV/AIDS.

80 patients who came to the hospital for physical examination during the same period were enrolled as the control group. These were diagnosed as having no hepatitis virus infection by virus serology and liver biochemical indices, with normal liver function and no abnormal abdominal ultrasound examination.

All enrolled patients (in both cohorts) provided informed consent as approved by the research ethical committee of Guizhou Medical University and Affiliated Hospitals (No. 2021183) and conducted in accordance with the 1964 Helsinki Declaration and its later amendments or comparable ethical standards.

### Laboratory analysis

Following at least 12 h of fasting, 10 mL of whole blood was obtained from each member of the study population by vein puncture and collected in sterile tubes. Serum was then separated from blood cells within 20 min of collection by centrifugation at 1500 × g for 10 min at room temperature. To preserve the serum components, the sera were quickly aliquoted into micro-tubes and snap frozen at -80℃ to await further analysis. An automatic enzyme label analyzer (Shenzhen Akcome Company, China) was used to measure the serum levels of TET3; assays were performed with an enzyme-linked immunosorbent assay kit (Jiangsu Boshen Biotechnology Co., Ltd., China).

Serum samples were tested retrospectively for both hepatitis B virus (HBV) DNA and hepatitis B surface antigen (HBsAg) levels. HBsAg levels were quantified with an automatic immunofluorescence analyzer (Atecom Technology Co., Ltd., China). HBV DNA levels were quantified using a AGS4800 real-time fluorescent quantitative PCR using a 0.2 mL protocol (Anyu Biotechnology Co., Ltd., China) with a detection limit of 10 IU/mL.

A Roche Beckman Coulter AU5800 automatic biochemical analyzer was used to detect the levels of ALT, AST (IFCC, FUJIFILM Wako Pure Chemical Corporation) and other indicators. Platelet counts (PLT) were detected in anti-coagulated whole blood with a Sysmex XN9000 automatic blood analyzer.

### Calculation of FIB4 and APRI indices

FIB-4 and APRI indices were calculated according to the following formulas: FIB-4 = age (years) × AST[U/L]/(platelet counts [10^9^/L] × (ALT[U/L])1/2); APRI = (AST level/upper limit of normal of AST)/platelet counts [10^9^/L] × 100^23^. The upper limit for a normal AST level was 35 U/L.

### Statistical analysis

Continuous variables that were distributed normally are expressed as means and standard deviations (Kolmogorov-Smirnow normality test) whereas those that were not normally distributed are expressed as the median (range) and processed by the Mann–Whitney *U* test. Categorical parameters are presented as frequencies. To determine independent risk factors of liver fibrosis and cirrhosis, variables with *P* < 0.05 in the univariate logistic analysis were subsequently included in multivariate logistic regression. The diagnostic values for positive predictive value (PPV), negative predictive value (NPV) and the cut-off values for serum TET3 were assessed by ROC curve analysis. Associations between the variables were tested using calculations of correlation coefficients (Spearman’s correlation). Statistical analysis was performed using SPSS, version 19 (SPSS, Inc., Chicago, IL, United States). Statistical significance was assigned to *P* values < 0.05.

### Ethics approval

All enrolled patients (in both cohorts) provided informed consent and approved by the research ethical committee of Guizhou Medical University and Affiliated Hospitals (approval number:No. 2021183).

### Informed consent

Investigators have to obtain informed consent before enrolling participants in Clinical trials.

## Results

### Patient and control cohorts

The baseline characteristics of 212 patients with CLD are shown in Table 1. Of these, 140 patients were diagnosed with chronic hepatitis (56.43% male with a mean age (± SD) of 45.34 ± 12.91 years). In total, 72 patients were diagnosed with cirrhosis (76.39% male with a mean age of 48.94 ± 12.59 years). Furthermore, 77.86% (109/140) of patients with hepatitis and 90.28% (65/72) of patients with cirrhosis were positive for HBsAg. According to the diagnostic criteria of liver fibrosis^21^, of the 140 cases with chronic hepatitis, 38 (27%) were classified as non-fibrosis while 102 (73%) were classified as fibrosis (Table 2). There was no significant age difference between the non-fibrosis group and the fibrosis group (mean age: 41.92 years *vs.* 46.62 years). Blood tests were performed for all enrolled patients so that we could determine platelet count, AST, ALT, ARPI score and FIB-4 index.

**Table 1:** Baseline Characteristics of the 212 patients with chronic liver disease.

	Control (n = 80)	Chronic liver disease	
		Hepatitis (n = 140)	Cirrhosis(n = 72)
Average age, years (range)	41.86(20,70)	45.34(15,86)	48.94(19,74)^a^
Gender			
Male (%)	53(66.25)	79(56.43)	55(76.39)
Female (%)	27(33.75)	61(43.57)	17(23.61)^b^
Serum ALT level (U/L),(Median, range)	20.70(15.33,25.88)	28.25(17.38,50.60)^a^	29.25(20.73,48.50)^a^
Serum AST leve (U/L),(Median, range)	19.90(16.98,22.68)	26.75(20.65,41.68)^a^	35.25(24.13,54.33)^ab^
APRI score (Median, range)	0.24(0.18,0.31)	0.49(0.34,0.77)^a^	1.41(0.64,2.62)^ab^
PLT(× 10^9^/L) (Median, range)	232.00(197.75,281.50)	174.50(135.25,213.75)^a^	88.00(57.25,129.75)^ab^
HBsAg (> 0.4 IU/mL)			
Negative (%)	80(100)	31(22.14)	7(9.72)
Positive (%)	0(0)	109(77.86)^a^	65(90.28)^ab^
FIB-4 Index (Median, range)	0.75(0.57,0.98)	1.46(0.93,2.22)^a^	4.04(1.89,7.86)^ab^
TET3 ng/mL	127.58(96.99,163.7)	268.10(133.97,900.56)^a^	1148.09(362.64,1451.11)^ab^

^a^*P* < 0.05 versus control; ^b^*P* < 0.05 versus hepatitis.

Abbreviations:ALT, alanine aminotransferase; AST, aspartate aminotransferase; FIB-4, fibrosis-4; APRI, AST to platelet ratio Index; PLT, platelet count; HBsAg, Hepatitis B surface antigen; TET3, Ten-eleven translocation protein 3.

**Table 2:** Comparison of the clinical characteristics of patients with and without non-fibrosis.

	Non-fibrosis (n = 38)	Fibrosis (n = 102)
Average age, years (range)	41.92(15,64)	46.62(20,86)
Gender		
Male(%)	17(44.74)	62(60.78)
Female(%)	21(55.26)	40(39.22)
Serum ALT level (U/L) (Median, range)	45.20(19.45,235.10)	25.50(16.48,41.50)^a^
Serum AST leve (U/L) (Median, range)	42.05(24.53,90.40)	24.80(20.00,35.03)^a^
APRI score (Median, range)	0.71(0.36,1.23)	0.46(0.31,0.70)^a^
PLT(× 10^9^/L) Mean ± SD	201.33 ± 60.89	167.88 ± 55.07^a^
HBsAg (> 0.4 IU/mL)		
Negative (%)	18(47.34)	13(12.75)
Positive (%)	20(52.63)	89(87.25)^a^
FIB-4 Index (Median, range)	1.33(0.83,1.83)	1.49(0.96,2.26)
TET3 ng/mL (Median, range)	117.73(71.37,174.36)	648.66(154.62,1040.71)^a^

^a^*P* < 0.05 versus Non-fibrosis.

*ALT*, alanine aminotransferase; *AST*, aspartate aminotransferase; *FIB-4*, fibrosis-4; *APRI*, AST to platelet ratio Index; *PLT*, platelet count; *HBsAg*, Hepatitis B surface antigen; *TET3*, Ten-eleven translocation protein 3.

### Serum TET3 level in chronic liver disease

**Figure 1:**
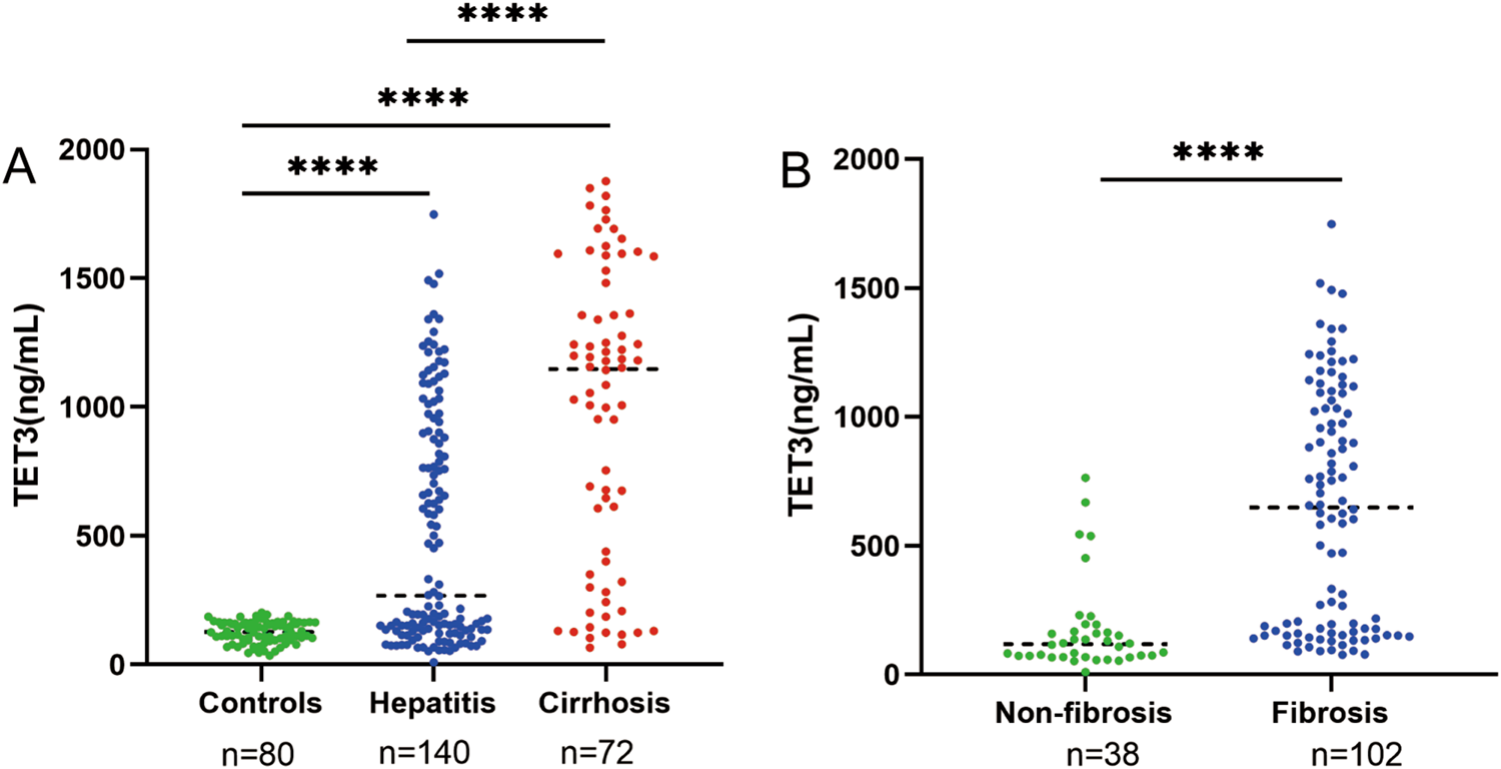
Serum TET3 level in chronic liver disease. (**A**) Comparison of serum TET3 levels in hepatitis, liver cirrhosis and controls; (**B**) Comparison of serum TET3 levels between the patients of fibrosis and non-fibrosis with hepatitis.

Serum TET3 level in cirrhosis cases (median 1148.09, range 362.64–1451.11 ng/mL) was significantly higher than that in hepatitis (median 268.10, range 133.97–900.56 ng/mL) and controls (median 127.58, range 96.99–163.7 ng/mL), respectively. The serum TET3 level in chronic hepatitis was significantly higher than that in controls (*P* < 0.0001), Fig. 1A. Considering the two major groups of cases, namely non-fibrosis (median 117.73, range 71.37–174.36 ng/mL) and fibrosis (median 648.66, range154.62–1040.71 ng/mL) subjects, the TET3 levels in fibrosis were significantly higher in non-fibrosis (*P* < 0.001), Fig. 1B. There was no significant difference in TET3 level between the non-fibrosis and the controls.

### Serum levels of TET3 and clinical indicators

**Figure 2:**
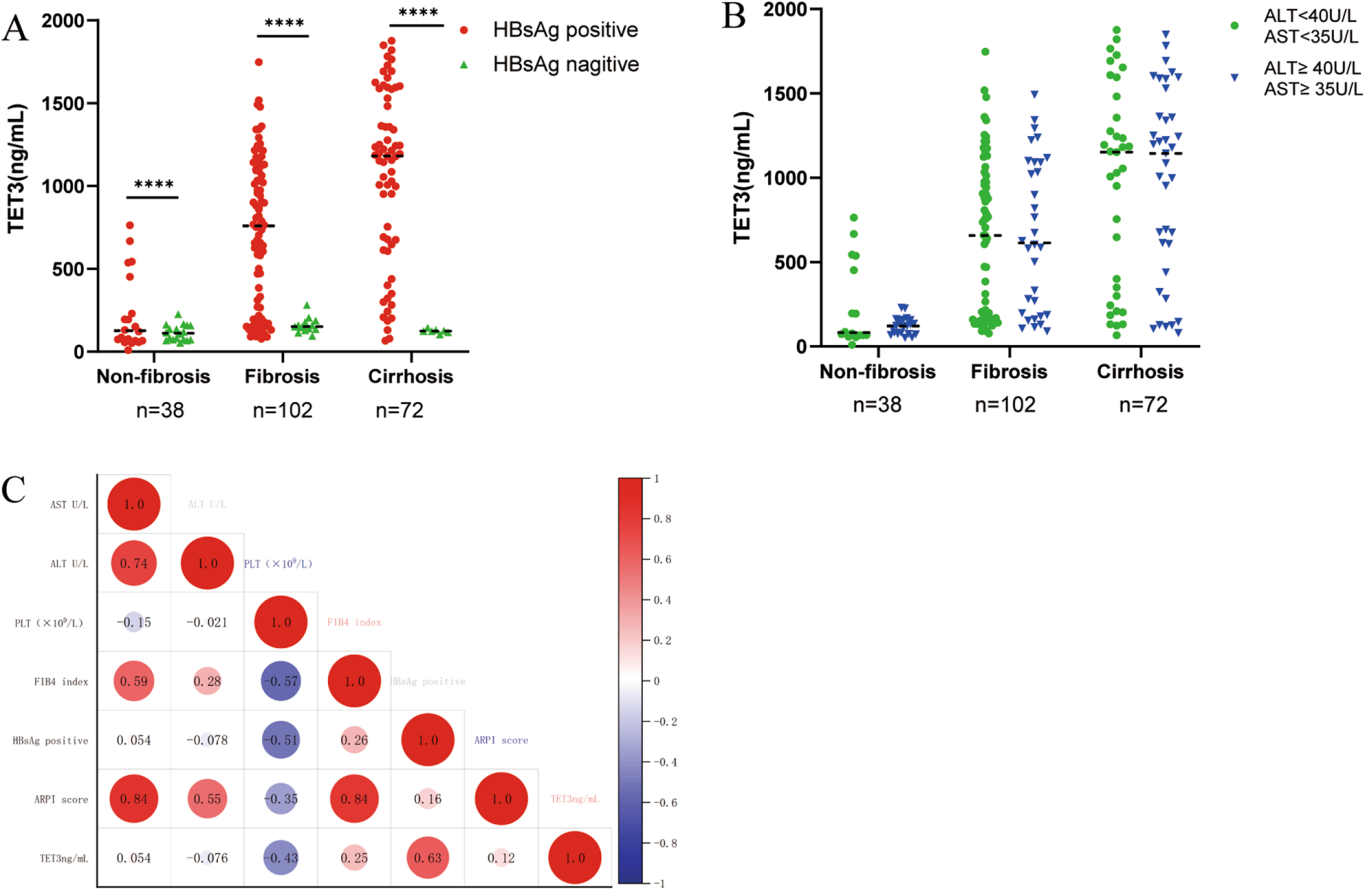
Serum levels of TET3 and Clinical indicators. (**A**) When HbsAg were more than or less than 0.4 IU/mL, TET3 levels were observed between non-fibrosis, fibrosis and cirrhosis groups; (**B**) When AST and ALT were more than or less than 35 U/L or 40U/L, TET3 levels were observed between non-fibrosis, fibrosis and cirrhosis groups; (**C**) Correlation of TET3 level and clinical indicators in all patients.

Statistical analysis was performed for the serum levels of TET3 and several key clinical indicators (AST, ALT and HBsAg). We found that the patients with non-fibrosis, fibrosis and cirrhosis with HBsAg were > 0.4 IU/mL and that the levels of TET3 were significantly higher than those with HBsAg < 0.4 IU/mL, which was positively correlated (*r* = 0.63,* P* < 0.0001) with serum TET3 levels (Fig. 2A,C).

In line with our inclusion criteria, levels of aminotransferase were higher in cases than in normal controls (Table 1). When AST and ALT were more than or less than 35 U/L or 40 U/L, there was no significant difference in TET3 levels when compared between the non-fibrosis, fibrosis and cirrhosis groups. Correlation analysis showed that these were not correlated with serum TET3 levels (AST: *r* = 0.054; ALT: *r* = 0.076; *P* > 0.05; Fig. 2B,C). We also found that serum TET3 levels were positively correlated with FIB4 index and APRI score (*r* = 0.25; 0.12, *P* < 0.001, respectively) and negatively correlated with platelet count (*r* = −0.53, *P* < 0.001), Fig. 2C.

### Univariate and multivariable analyses for TET3

Non-invasive markers such as the FIB-4 index^24^ and the APRI score^25^ can be used to predict hepatic fibrosis. Therefore, we included the FIB-4 index and APRI score in our univariate and multivariate analyses. According to univariate analysis, platelet count, FIB-4 index and TET3 level were identified as independent predictors for liver fibrosis. Multivariable logistic analysis thus included platelet count, FIB-4 index and TET3 level (Table 3). Based on the multivariate analysis results, the odds ratio (OR) for FIB-4 index was 1.646 (95% CI 1.014–2.672, *P* = 0.044) and the OR for TET3 level was 1.006 (95% CI 1.004–1.008, *P* < 0.0001).

**Table 3:** Univariate and multivariable analyses for liver fibrosis and cirrhosis.

	Fibrosis (n = 220)				Cirrhosis (n = 292)			
	Univariate Analysis		Multivariate Analysis		Univariate Analysis		Multivariate Analysis	
	*OR* (95% *CI*)	*P* Value	*OR* (95% *CI*)	*P* Value	*OR* (95% *CI*)	*P* Value	*OR* (95% *CI*)	*P* Value
Age (yr)	1.031(1.009–1.054)	0.007	1.018(0.983–1.054)	0.330	1.030(1.009–1.053)	0.006	0.987(0.949–1.028)	0.534
Gender (n)	1.063(0.619–1.826)	0.825		NA	2.157(1.175–3.958)	0.013	2.396(0.965–5.951)	0.060
ALT (U/L)	0.996(0.992–1.000)	0.052		NA	1.000(0.998–1.003)	0.727		**NA**
AST (U/L)	0.999(0.993–1.004)	0.617		NA	1.006(1.002–1.010)	0.007	0.994(0.968–1.021)	0.682
PLT (× 10^9^/L)	0.983(0.978–0.989)	< 0.0001	0.993(0.985–1.002)	0.124	0.972(0.965–0.979)	< 0.0001	0.985(0.975–0.996)	0.008
APRI score	1.312(0.947–1.818)	0.103		NA	2.120(1.604–2.802)	< 0.0001	0.927(0.368–2.335)	0.872
FIB-4 index	2.373(1.625–3.465)	< 0.0001	1.646(1.014–2.672)	0.044	1.772(1.494–2.102)	< 0.0001	1.533(1.019–2.306)	0.040
TET3 (ng/mL)	1.007(1.004–1.009)	< 0.0001	1.006(1.004–1.008)	< 0.0001	1.002(1.002–1.003)	< 0.0001	1.002(1.001–1.003)	< 0.0001

NA: not adopted.

We also analyzed the predictive significance of these parameters for cirrhosis. Multi variate analysis, which included age, gender, AST, platelet count, APRI score, FIB-4 index and TET3 level, showed that platelet count, FIB-4 index and TET3 level were independent risk factors for cirrhosis with ORs of 0.985 (95% CI 0.975–0.996, *P* = 0.008), 1.533 (95% CI: 1.019–2.306, *P* = 0.040) and 1.002 (95% CI 1.001–1.003, *P* < 0.0001), respectively (Table 3). Thus, we chose TET3 level and FIB-4 index to predict liver fibrosis and cirrhosis. The probabilities of the TET3-FIB-4 index model were calculated using the following formula: (liver fibrosis: ŷ = 1/[1 + exp. (5.859–(0.02 × TET3 (ng/mL))–(0.477 × FIB-4 index)]; cirrhosis: ŷ = 1/[1 + exp. (9.85–(0.03 × TET3 (ng/mL))–(2.71 × FIB-4 index )]).

### Positive predictive value, negative predictive value and the accuracy of the biomarker

**Figure 3:**
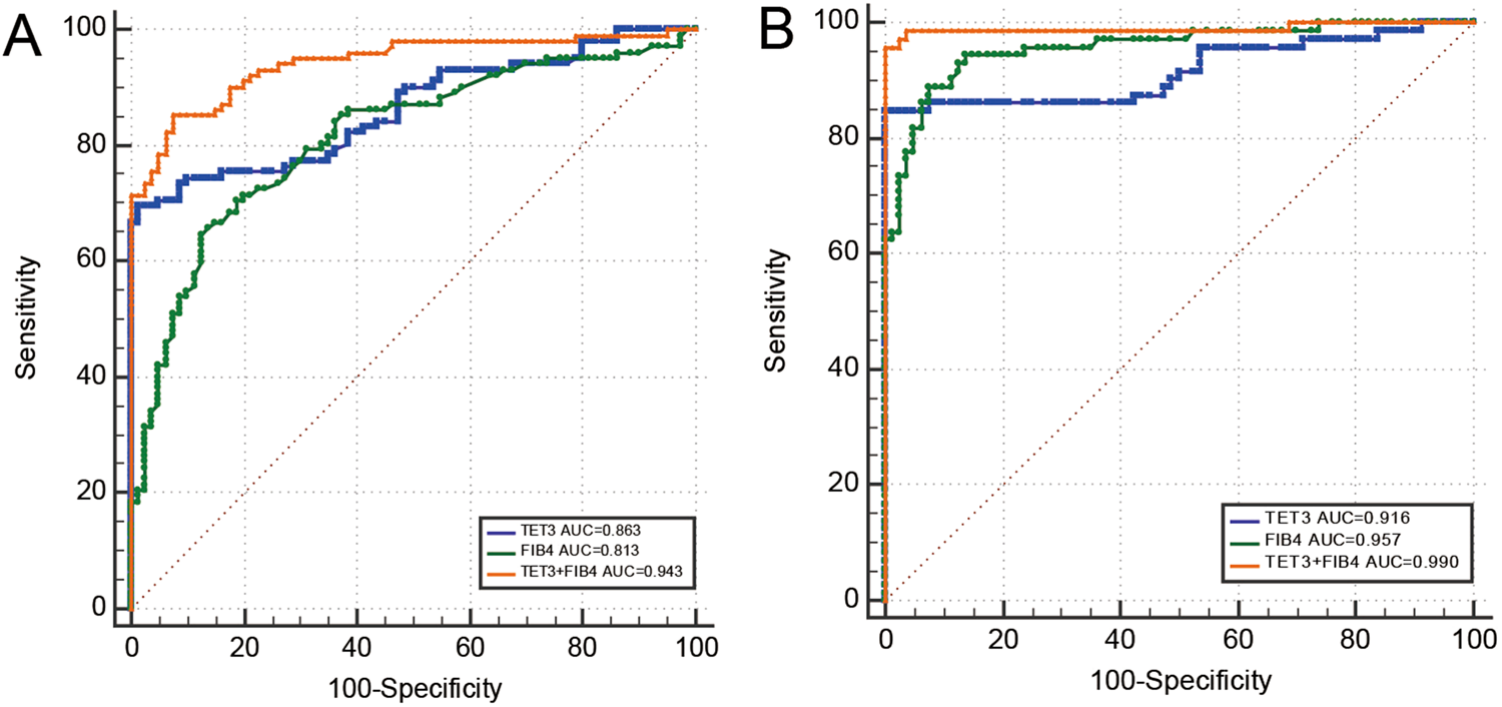
ROC analysis of serum TET3 and fibrosis and cirrhosis. (**A**) ROC curves for TET3, FIB-4 index, combination of TET3 and FIB-4 index for predicting Liver fibrosis; (**B**) ROC curve for TET3, FIB-4 index, combination of TET3 and FIB-4 index for predicting Liver cirrhosis. (**C**) ROC curve for TET3 for predicting liver fibrosis in chronic hepatitis B.

We generated an ROC curve to determine the positive predictive value and negative predictive value for TET3 and FIB-4 index at the optimal cutoff value. Area under the ROC (AUROC) curve analysis revealed that the TET3 level (AUROC = 0.863; 95% CI 0.804–0.909; *P* < 0.0001) for predicting the risk of liver fibrosis was higher than the FIB-4 index (AUROC = 0.813; 95% CI 0.749–0.867; *P* < 0.0001), Fig. 3A, Table 4. With an optimal cut-off value of 194.41 ng/mL, TET3 level had a high positive predictive value (PPV) of 98.60% and a negative predictive value (NPV) of 71.80% for predicting the risk of liver fibrosis. The optimal cut-off value of the FIB-4 index was 1.19, with a PPV of 86.80% and a NPV of 66.00% for predicting the risk of liver fibrosis. Our multivariate model featuring both TET3 level and FIB-4 index had a higher AUROC of 0.943 (95% CI 0.899–0.972) when compared with each diagnostic tool alone; this was statistically significant (*P* < 0.0001; Table 4). TET3-FIB-4 index cut-off value of 0.44 had a high PPV of 93.50% for ruling in patients with liver fibrosis.We took whether there was hepatitis B virus infection as the cause, and perform ROC curve analysis on chronic hepatitis B patients, found that the AUROC values of TET3 when used to predict the risk of liver fibrosis in patients with chronic hepatitis B was 0.852 (95% CI 0.771–0.913; *P* < 0.0001; Fig. 3 supplementary). With an optimal cut-off value of 543.79 ng/mL, TET3 level had a high PPV of 96.70% .

**Table 4:** Diagnostic test characteristic of TET3, FIB-4 index and multivariable model in detecting liver fibrosis and cirrhosis.

Model	Fibrosis					Cirrhosis				
	AUROC(95%CI)	*P* value	Cut-point	NPV	PPV	AUROC(95%CI)	Cut-point	*P* value	NPV	PPV
TET3(ng/mL)	0.863(0.804–0.909)	< 0.0001	194.41	71.80	98.60	0.916(0.860–0.955)	201.62	< 0.0001	87.90	100
FIB-4 index	0.813(0.749–0.867)	< 0.0001	1.19	66.00	86.80	0.957(0.911–0.983)	1.84	< 0.0001	90.20	91.40
TET3 + FIB-4 index	0.943(0.899- 0.972)	< 0.0001	0.44	83.10	93.50	0.990(0.958–0.999)	0.52	< 0.0001	96.40	100

NPV: Cut-point was derived from the Youden’s index, NPV: Negative predictive value, PPV: Positive predictive value.

Next, we investigated the predictive value of liver cirrhosis in 72 patients. The results remained significant in the cirrhosis cohort, Table 4. With an optimal cut-off value of 201.62 ng/mL, TET3 level had a high PPV of 100% and a NPV of 87.90% for predicting the risk of liver cirrhosis. The optimal cut-off value of the FIB-4 index was 1.84, with a PPV of 91.40% and a NPV of 90.20% for predicting the risk of liver cirrhosis. TET3 level coupled with the FIB-4 index had a higher AUROC of 0.990 (95% CI 0.958–0.999, Fig. 3B). TET3-FIB-4 index cut-off value of 0.52 had a high PPV of 100% for ruling in patients with liver cirrhosis.

## Discussion

In recent years, the incidence and mortality of chronic liver diseases have increased worldwide, thus creating a significant medical burden^26^. Liver fibrosis is a significant lesion of chronic liver disease. Most patients with chronic hepatitis have differing degrees of liver fibrosis; the early and middle stages of liver fibrosis can be reversed, at least to some extent^27^. Therefore, the prediction and early diagnosis of fibrosis are particularly important for the course and efficacy of the entire disease. At present, liver biopsy is considered to be the "gold standard" to evaluate the degree of liver fibrosis; however, biopsy is associated with trauma and potential complications, thus restricting the clinical development of this technique. Many scholars are committed to the non-invasive diagnosis of liver fibrosis, mainly by serum biochemical detection and physical imaging technology, such as liver fibrosis serum (direct and indirect serum) markers, ultrasound Doppler, liver elastography detection and other physical technologies^28^. Due to the fact that the onset of liver fibrosis is insidious and the early symptoms are not obvious, the lack of predictability makes the prognosis and patient stratification difficult. So far, there is no specific and effective non-invasive diagnostic index.

TET3 is one of the key enzymes in DNA demethylation and oxidizes 5mC to 5hmC. Studies have shown that TET3 can be expressed in liver tissues and is related to the occurrence and development of a variety of liver diseases^29–31^. TET3 could be a reliable biomarker for the establishment of early fibrosis in CLD. In the current study, we tested the serum levels of TET3 in 212 patients with CLD. The results showed that serum TET3 levels played no significant role in the diagnosis of chronic hepatitis (the non-fibrotic stage); however, this biomarker could be used to distinguish between fibrosis and cirrhosis. When the disease progresses into fibrosis, TET3 is valuable because it allows us to separate cases without fibrosis from those with fibrosis. Univariate and multivariate logistic analyses showed that TET3 levels are an independent predictor of liver fibrosis and cirrhosis (*P* < 0.0001). After accepting TET3 as a reliable biomarker, we plotted an ROC curve to show that the AUROC values of TET3 when used to predict the risk of liver fibrosis and cirrhosis were 0.916 and 0.863, respectively. With the cut-off value of TET3 set to 194.41 ng/mL, the positive predictive value was 98.60% and the negative predictive value was 71.80% for predicting the risk of liver fibrosis. With the cut-off value of TET3 set to 201.62 ng/mL, the positive predictive value was 100% and the negative predictive value was 87.90% for predicting the risk of cirrhosis .

Hepatitis B virus (HBV) is the most common factor leading to liver injury in China^32^. HBV is a hepatophilic virus with strong infectivity and mainly exists in hepatocytes, causing damage to hepatocytes that leads to inflammation, necrosis and fibrosis. All of our cases were tested for hepatitis B surface antigen (HBsAg); we found that the levels of TET3 were significantly higher in patients with an HBsAg greater than 0.4 IU/mL than in patients with an HBsAg < 0.4 IU/mL; these two factors were positively correlated (*r* = 0.63,* P* < 0.0001) with the serum level of TET3. These results indicate that HBV infection may cause an increase in the serum levels of TET3 although the mechanisms underlying this effect need to be investigated. We also found that TET3 was a good predictor of liver fibrosis in patients with chronic hepatitis B, with a high AUROC of 0.852 and PPV of 96.70% .

ALT and AST are the most commonly used biochemical indices for detecting liver function. When hepatocytes are damaged or become necrotic, a large amounts of AST and ALT are released into the blood; the levels of these factors are closely related to the degree of hepatocyte damage^33^. We found that when AST and ALT were more than or less than 35 U/L or 40 U/L, there was no significant difference in the serum levels of TET3 in non-fibrosis, fibrosis, and cirrhosis. Correlation analysis showed that these parameters were not correlated with serum TET3 levels (AST *r* = 0.054 and ALT *r* = 0.076; *P* > 0.05). This suggested that the increase of serum TET3 levels in patients with liver fibrosis and cirrhosis is not related to ALT and AST, and also indicates that TET3 may be a secreted protein; the increase of TET3 in the peripheral blood may not be released into the blood due to the destruction of hepatocytes.

In medicine, a biomarker is a measurable trait that reflects the severity or presence of certain disease states. In other words, a biomarker is an indicator of a particular pathological condition or some other physiological state of an organism. Many serological biomarkers have been used to diagnose and evaluate liver fibrosis, such as hyaluronic acid^34^, CTGF^35^, FIB-4 index^24^, APRI score^25^ and Forns index^36^. FIB-4 is a non-invasive, simple, rapid, and low-cost test that can assess the grade of liver fibrosis^37,38^. This particular index combines AST, ALT, platelet count, and age and was originally used for staging liver fibrosis in patients with hepatitis C virus and HIV. More recently, this index has been used to quantify fibrosis grade in various liver diseases, including HCV or HBV infection, ALD, and NAFLD^24^. However, FIB-4 is susceptible to changes in ALT, AST, platelet count or patient age, thus resulting in lower accuracy when evaluating the degree of liver fibrosis^39^. In this study, univariate and multivariate logistic analyses showed that FIB-4 index was an independent predictor of liver fibrosis and cirrhosis (Table 3). The AUROC values of the FIB-4 index when used to predict liver fibrosis and cirrhosis were 0.813 and 0.957, respectively. The FIB-4 index had lower predictive efficacy for liver fibrosis than TET3 level, but higher predictive efficacy for liver cirrhosis than TET3 level.

The progressive development of fibrosis to end-stage liver cirrhosis is influenced by many factors; thus, the lack of predictability makes disease prognosis and patient stratification very difficult. To date, no single biomarker or scoring system has provided optimal sensitivity and specificity for detecting significant liver fibrosis, especially in the early stages. Multivariate combination is a key trend in current development. Although TET3 levels and the FIB-4 index have their own limitations, they can complement each other to increase their discriminatory power to predict liver fibrosis and cirrhosis. In our study cohort, we demonstrated that a model including TET3 and FIB-4 index exhibited a highly promising PPV for detecting stages of liver fibrosis and cirrhosis (93.50% and 100%, respectively) when compared with each diagnostic tool alone.

Importantly, our study is the first to show that serum TET3 levels can be detected by simple and rapid serological methods. Our research demonstrated that TET3 is a novel and reliable tool for predicting liver fibrosis and cirrhosis. In particular, the combination of TET3 and FIB-4 index could enhance discriminatory power to predict liver fibrosis and cirrhosis when compared to TET3 or the FIB-4 index alone. However, this study still has some limitations that need to be considered. For example, the sample size was small. Furthermore, the chronic liver diseases included in this study included drug-induced liver injury, ALD, NAFLD and liver disease associated with hepatitis virus infection. Unfortunately, the stage of liver fibrosis and hepatitis was not carried out. Hence, further studies with a larger number of cases from different etiologies are recommended to clarify the specific role of TET3 for the detection of fibrosis in such patients.

## Conclusion

TET3 is related to the development of liver fibrosis and cirrhosis. TET3 is a novel, rapid and effective predictive tool for liver fibrosis. The accuracy of the TET3-FIB-4 model to predict liver fibrosis and cirrhosis was significantly better than that of TET3 or the FIB-4 index alone.

## Supplementary Information

Supplementary Information 1.

## Data Availability

All data is available from the corresponding author.
