# Supplementary figures and images for: TET3 as a non-invasive screening tool for the detection of fibrosis in patients with chronic liver disease
Source: Sci Rep. 2023 Apr 19;13:6382. doi: 10.1038/s41598-023-33564-7 (PMC10115894; doi:10.1038/s41598-023-33564-7)

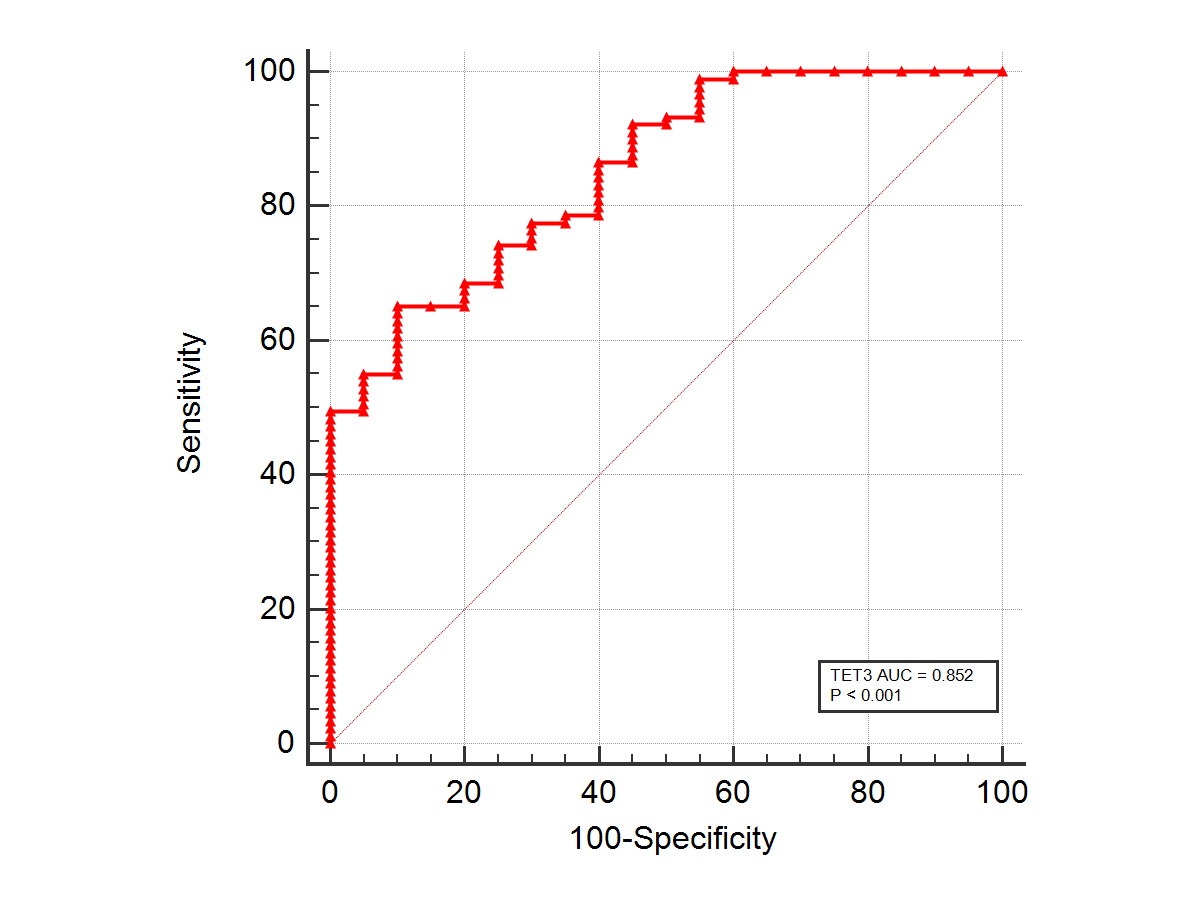

Supplement: Supplementary file 1 — Supplementary Information 1. [file 41598_2023_33564_MOESM1_ESM.tif]
